# Association between estimated glucose disposal rate and prediabetes reversion and progression: a nationwide cohort study of middle-aged and elderly people in China

**DOI:** 10.3389/fendo.2025.1500993

**Published:** 2025-03-21

**Authors:** Xin Huang, Shiming He, Chao Wang, Guoan Jian, Kun Jiang, Zihao Lu, Wei Wang, Guotai Sheng, Yang Zou

**Affiliations:** ^1^ Jiangxi Cardiovascular Research Institute, Jiangxi Provincial People’s Hospital, The First Affiliated Hospital of Nanchang Medical College, Nanchang, Jiangxi, China; ^2^ Jiangxi Provincial Geriatric Hospital, Jiangxi Provincial People’s Hospital, The First Affiliated Hospital of Nanchang Medical College, Nanchang, Jiangxi, China

**Keywords:** estimated glucose disposal rate, prediabetes to normoglycaemia, diabetes, cohort study, Chinese

## Abstract

**Objective:**

Prediabetes is a chronic condition characterized by elevated blood glucose levels that are not yet high enough to be classified as diabetes. It is particularly prevalent among middle-aged and elderly populations. This study aims to investigate the association between a novel marker of insulin resistance-the estimated glucose disposal rate (eGDR)-and the reversion of prediabetes to normoglycaemia or progression to diabetes in a Chinese population.

**Methods:**

This prospective cohort study utilized baseline data from the 2011 China Health and Retirement Longitudinal Study involving 2,600 prediabetic participants aged 45 years and older, along with follow-up data from 2015. The study’s endpoints were defined according to the American Diabetes Association criteria, including maintenance of the prediabetic state, reversion to normoglycaemia, or progression to diabetes. Multivariable Cox regression models and restricted cubic spline regression were used to assess the association between eGDR and the reversion or progression of prediabetes in middle-aged and elderly populations, followed by stratified analyses to explore potential population-specific dependencies.

**Results:**

Over a median follow-up period of 4 years, 1,615 (62.1%) participants remained in the prediabetic state, 586 (22.5%) reverted to normoglycaemia, and 399 (15.3%) progressed to diabetes. In multivariable Cox regression analyses, our results indicated that eGDR was positively associated with the reversion of prediabetes to normoglycaemia [Hazard Ratio = 1.14, 95% Confidence Interval: 1.05, 1.23], and negatively associated with the progression of prediabetes to diabetes (HR = 0.81, 95% CI: 0.70, 0.93). Restricted cubic spline analysis revealed a nonlinear, L-shaped association between eGDR and the reversion of prediabetes to normoglycaemia, with segmented Cox regression identifying an eGDR threshold of 6.81 as the point of significant change in the likelihood of prediabetes reversion.

**Conclusion:**

This prospective cohort study among middle-aged and elderly Chinese populations suggested that higher eGDR promoted the reversion of prediabetes and provided a protective effect against its progression to diabetes.

## Background

Diabetes is a metabolic disorder characterized by chronic hyperglycemia and is closely associated with increased mortality rates (1). According to data from the International Diabetes Federation, the global prevalence of diabetes was 10.5% in 2021, and it is projected to rise to 12.2% over the next 24 years (2). Given the large and growing number of individuals with diabetes, there is an urgent need to develop effective strategies for the prevention of this disease. Prediabetes is an intermediate stage between normoglycaemia and diabetes, and it is used to identify individuals at high risk for future diabetes (3). Studies have shown that prediabetes represents the optimal stage for intervention in the worsening trajectory of blood glucose levels (4). Several completed randomized controlled trials have demonstrated that lifestyle and pharmacological interventions can effectively control the progression of prediabetes, thereby reducing the incidence of diabetes and cardiovascular complications (5–7). Even a temporary reversion to normoglycaemia in individuals with prediabetes is significantly associated with a reduced incidence of diabetes (8). Therefore, early identification of modifiable factors influencing the reversion of prediabetes to normoglycaemia or its progression to diabetes is crucial for reducing the burden of diseases related to impaired glucose metabolism.

IR is a pathophysiological condition characterized by reduced responsiveness of target organs or tissues to insulin, leading to impaired glucose utilization. It is a significant risk factor for the development of diabetes and cardiovascular diseases (9). Early monitoring and control of IR are vital for the reversion or progression of prediabetes. The Hyperglycemic-Euglycemic Glucose Clamp (HEGC) technique is considered the gold standard for assessing IR, and evaluating insulin sensitivity by measuring the Glucose Disposal Rate (GDR) (10). However, HEGC is invasive, relatively complex, and time-consuming, making it impractical for routine clinical practice (10, 11). To address this limitation, researchers have derived an estimated GDR (eGDR) based on HEGC data, calculated as 21.158 − [0.09 × waist circumference (WC, cm)] − [3.407 × hypertension (yes = 1, no = 0)] − [0.551 × hemoglobin A1c (HbA1c, %)], which can be utilized for assessing insulin sensitivity (12). To date, eGDR as a measure of IR has been validated in numerous related studies across various ethnic groups (13–19). In summary, eGDR can serve as a surrogate marker for IR in assessing various study outcomes in both diabetic and non-diabetic patients. Compared to HEGC, eGDR holds greater potential as a tool for epidemiological studies and clinical practice applications. It should be noted that although a substantial body of research has provided evidence for the use of eGDR as a surrogate for IR in both diabetic and non-diabetic populations, it remains unclear whether eGDR can be utilized for risk assessment of diabetes as a study outcome. Additionally, we lack understanding of the role of eGDR in the transition from hyperglycemia to normoglycemia. Addressing these issues will facilitate a deeper understanding of the associative relationship between eGDR and glucose metabolism, which is crucial for enhancing the application and awareness of eGDR. To address this issue, this study aims to explore the association between eGDR and the reversion or progression of prediabetes using data from the large longitudinal China Health and Retirement Longitudinal Study (CHARLS) cohort in China.

## Methods

### Study design and data source

CHARLS is a nationally representative longitudinal survey conducted in mainland China, primarily targeting middle-aged and elderly populations. The survey assesses demographic background, health status and function, social and economic conditions, and retirement information (20). The detailed study design is summarized in the online **Supplementary Methods**. In brief, the nationwide CHARLS survey was conducted from 2011-2012, employing geographic information system software and a multistage probability sampling method to construct the sample set. A total of 17,708 individuals were recruited from 28 provinces, 150 counties, and 450 villages/communities. Follow-up surveys have been conducted every 2-3 years, and to date, CHARLS has released five waves of longitudinal survey data. It should be noted that multistage probability sampling is a step-by-step sampling method particularly suited to large-scale surveys. Its core concept involves dividing the population into multiple stages, progressively narrowing the scope until the final sample is selected.

**Figure 1:**
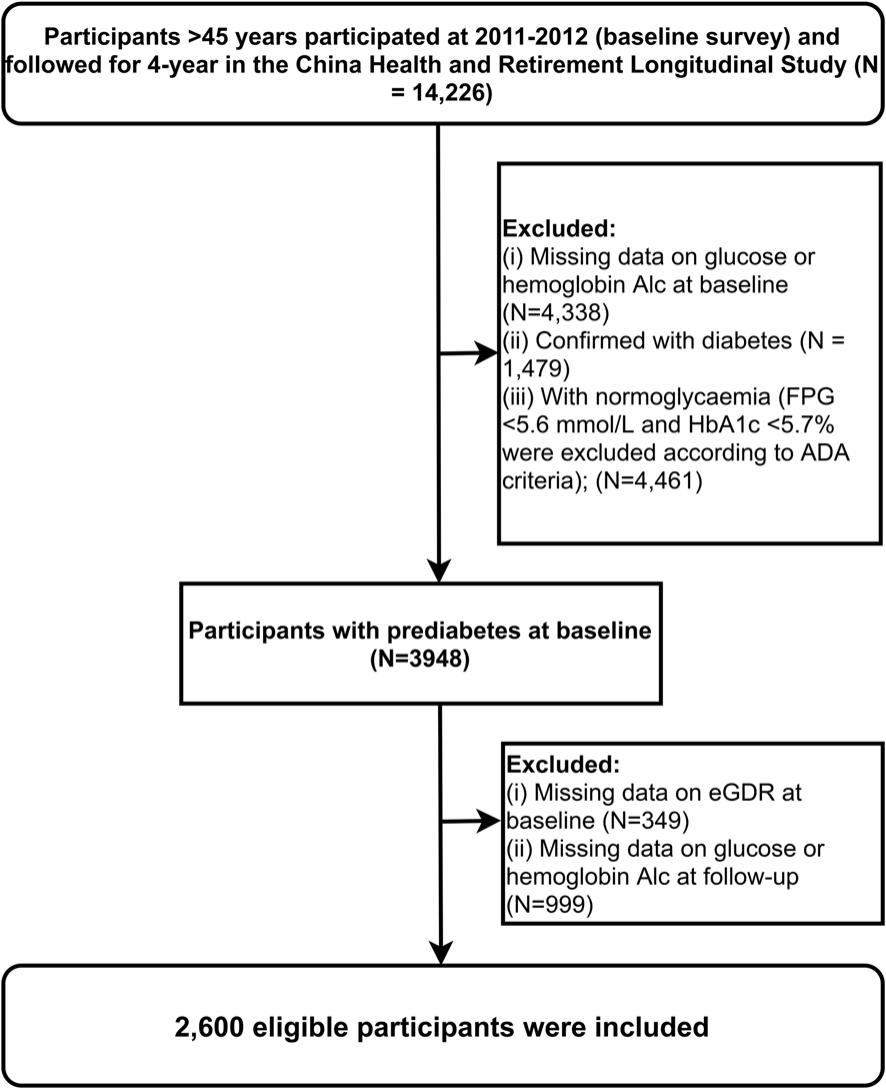
Flow chart of study participants.

Specific information about the CHARLS cohort survey questionnaire has been described elsewhere (20). For this study, we utilized data from 2011-2012 (Wave 1) and 2015-2016 (Wave 3) CHARLS surveys (N=14,226) for statistical analysis, as these two waves included blood sample information necessary for evaluating glucose metabolism. Based on the study objectives, the following exclusion criteria were applied: participants with missing glucose or HbA1c data at baseline (N=4,338) were excluded first, followed by those with normoglycaemia or diagnosed diabetes at baseline (N=1,479). Additionally, we excluded participants with uncertain hypertension status (N=11), missing WC data (N=338), and those without glucose or HbA1c measurements at follow-up (N=999). Ultimately, 2,600 individuals were included in the current study. The detailed study flow is illustrated in **Figure 1**.

### Ethical approval

This study was conducted in accordance with the Declaration of Helsinki and adhered to the STROBE reporting guidelines. The study protocol for the CHARLS project was approved by the Ethics Review Committee of Peking University (IRB00001052-11015). To ensure the protection of participants’ rights, all individuals involved in the CHARLS project were fully informed and provided written informed consent before the commencement of the study. Clinical trial number: not applicable.

### Baseline data collection

Baseline information was collected by trained interviewers through face-to-face computer-assisted personal interviews, using structured questionnaires to gather data on sociodemographic status, health-related factors, comorbidities, and laboratory measurements. Sociodemographic variables included gender, age, education level (below primary, primary schools, middle school, high school and above), marital status (married, other), and residence (rural, urban). Health-related factors included height, weight, WC, body mass index (BMI), systolic blood pressure (SBP), diastolic blood pressure (DBP), and smoking/drinking status (never, current, quit). Comorbidities included hypertension, cardiovascular disease (CVD), and stroke, with detailed diagnostic information provided in the online **Supplementary Methods**. Laboratory measurements were taken after participants had fasted for at least 8 hours and included serum creatinine (Cr), uric acid (UA), total cholesterol (TC), triglycerides (TG), high-density lipoprotein cholesterol (HDL-C), low-density lipoprotein cholesterol (LDL-C), glucose, and HbA1c.

### Definition of glycemic metabolic status

In the current study, we assessed glucose status based on the definition of impaired fasting glucose according to the American Diabetes Association criteria (21), which includes prediabetes, diabetes, and normoglycemia. Detailed information is presented in **Table 1**.

**Table 1:** Definition of diabetes, prediabetes and normoglycemia according to the American Diabetes Association criteria.

	Normoglycaemia	Prediabetes	Diabetes
ADA			
FPG	<5.6 mmol/L	5.6-6.9 mmol/L	≥ 7.0 mmol/L
HbA1c	<5.7%	5.7-6.4%	≥ 6.5%
Diagnosed with diabetes by another physician			Yes

HbA1c, hemoglobin A1c; FPG, fasting plasma glucose; ADA, American Diabetes Association;

for participants with random plasma glucose (RPG) measurements, RPG <7.8 mmol/L indicated normal glucose, while RPG >11.1 mmol/L indicated diabetes.

### Statistical analysis

Initially, baseline data were described, with continuous variables presented as means (standard deviations) or median (interquartile interval), and categorical variables as frequencies and percentages. Next, participants were grouped according to eGDR quartiles (Q1-Q4), and differences in baseline characteristics were analyzed using one-way ANOVA, Kruskal-Wallis H test, and chi-square test.

Univariable and multivariable Cox regression models were used to calculate Hazard Ratios (HRs) and corresponding 95% Confidence Intervals (CIs) to quantify the impact of eGDR on the reversion or progression of prediabetes. Before constructing the model, we first calculated variance inflation factors for all covariates to ensure that there was no multicollinearity among the covariates in the multivariate model constructed subsequently (**Supplementary Table S1**) (22). Several stepwise adjustment models were built based on clinical experience, relevant literature, collinearity diagnostics and Strengthening the Reporting of Observational Studies in Epidemiology guidelines. The crude model was unadjusted; Model 1 serves as a base-adjusted model that takes into account simple demographic and measurement information, including age, gender, marital status, height, and BMI; Model 2 was further adjusted for lifestyle habits (drinking status, smoking status) comorbidities (CVD and stroke) education, SBP, and DBP based on model 1; Model 3 served as the final model and was further adjusted for blood glucose, lipids (TG, HDL-C, LDL-C), Cr, and UA based on model 2. The validity of the proportional hazards hypothesis was tested using Schoenfeld residuals, and the Cox regression models of all clinical outcomes met the proportional hazards hypothesis.

To explore the dose-response relationship (linear or nonlinear) between eGDR and the reversion of prediabetes to normoglycaemia or progression to diabetes, we performed restricted cubic spline (RCS) analysis. Following Harrell’s recommendations (23, 24), four knots were placed at the 5th, 35th, 65th, and 95th percentiles of the eGDR distribution to minimize the potential impact of outliers, and a likelihood ratio test was used for nonlinear analysis (comparing the model with only a linear term against the model with linear and cubic spline terms). If the relationship was nonlinear, a recursive algorithm was employed to determine the inflection point (k-value) that maximized the model likelihood. A two-segment Cox regression model was then used on either side of the k-value to examine the association between eGDR and the reversion or progression of prediabetes. The specific method is to first run 3 models with inflection point equals Q1 (25% percentile), Q2 (50% percentile) and Q3 (75% percentile) within the range of Kmin and Kmax respectively to find out which quartile point gives the model with highest likelihood among the three models. Then we narrow down the Kmin and Kmax to the range of +/- 25% of the corresponding quartile point. By doing so, we narrow down the range of Kmin and Kmax 50% recursively each time until the specific value of the independent variable was identified, that if used as inflection point will give the segmented regression model highest likelihood.

After establishing the association between eGDR and glycemic metabolic status, we conducted stratified analyses to assess the modifying effects of gender, age, BMI, drinking/smoking status, education, marital status, hypertension, CVD, and stroke on the relationship between eGDR and the reversion or progression of prediabetes. Age groups were classified according to the World Health Organization’s standards (25), and BMI was categorized based on the recommendations of the Working Group on Obesity in China (26).

Finally, several sensitivity analyses were conducted to verify the robustness of the study findings: (1) The association between eGDR and the reversion or progression of prediabetes was validated in 1,227 participants diagnosed with diabetes, prediabetes, or normoglycaemia according to the Chinese expert consensus on prediabetes (27); (2) Given the potential competing risk relationship between reversion to normoglycaemia and progression to diabetes during follow-up, we further validated the association between eGDR and the reversion/progression of prediabetes in a competing risk model; (3) Model 3 was additionally adjusted for the quadratic term of age; (4) To account for missing baseline data (32 subjects missing SBP/DBP, 9 subjects missing height, 5 subjects missing weight, 12 subjects missing BMI, 12 subjects missing stroke, and 22 subjects missing CVD data; see **Supplementary Table S2**), multiple imputation using fully conditional specification was performed, and the main analyses were conducted on the complete dataset.

For all tests, statistical significance was set at *P* < 0.05. All analyses were conducted using R language version 3.4.3 and Empower(R) version 2.0.

## Results

### Baseline characteristics of participants

The current analysis included 2,600 middle-aged and elderly participants diagnosed with prediabetes, with an average age of 60 years. Among them, 1,197 were males, and 1,403 were females. The baseline characteristics of the study population were summarized according to eGDR quartiles (Q1-Q4). As shown in **Table 2**, participants with higher eGDR values (Q3, Q4) were more likely to be female, younger, and more educated, with lower height, weight, BMI, WC, UA, TC, TG, LDL-C, HbA1c, SBP, and DBP levels, and fewer comorbidities such as CVD and stroke, compared to those with lower eGDR values.

**Table 2:** Summary of baseline characteristics of the study population according to eGDR quartile group.

	eGDR quartiles				*P*-value
	Q1 (3.44-6.93)	Q2 (6.93-8.93)	Q3 (8.93-10.84)	Q4 (10.84-16.84)	
No. of subjects	650	650	650	650	
Age, years	60.50 (8.70)	61.85 (9.45)	57.58 (8.04)	58.06 (8.15)	<0.001
Height, m	1.58 (0.09)	1.57 (0.08)	1.59 (0.10)	1.57 (0.08)	<0.001
Weight, kg	67.64 (10.25)	55.52 (10.30)	62.76 (9.32)	52.25 (8.48)	<0.001
WC, cm	96.16 (6.40)	82.00 (7.97)	89.29 (6.71)	73.35 (12.62)	<0.001
BMI, kg/m^2^	27.03 (3.37)	22.41 (3.39)	24.72 (3.04)	21.14 (2.90)	<0.001
Cr, mg/dL	0.76 (0.67-0.89)	0.77 (0.67-0.89)	0.75 (0.64-0.87)	0.73 (0.64-0.85)	<0.001
UA, mg/dL	4.80 (1.28)	4.52 (1.29)	4.45 (1.17)	4.20 (1.17)	<0.001
TC, mg/dL	199.87 (176.68-224.90)	195.62 (174.36-218.72)	195.62 (173.20-220.75)	189.24 (166.62-216.11)	<0.001
TG, mg/dL	138.06 (98.23-195.36)	101.78 (73.68-148.68)	115.05 (81.42-167.04)	92.04 (67.26-131.64)	<0.001
HDL-C, mg/dL	44.85 (37.50-52.19)	52.19 (43.01-63.02)	48.33 (39.05-57.99)	53.93 (44.07-64.56)	<0.001
LDL-C, mg/dL	123.33 (99.74-146.91)	117.91 (95.49-136.47)	118.30 (99.84-140.72)	112.69 (93.65-135.60)	<0.001
Glucose, mmol/L	6.07 (0.47)	6.02 (0.40)	5.99 (0.47)	6.02 (0.37)	0.016
HbA1c, %	5.34 (0.40)	5.13 (0.41)	5.30 (0.41)	5.13 (0.40)	<0.001
SBP, mmHg	145.92 (19.46)	143.24 (20.09)	118.76 (10.60)	116.56 (11.50)	<0.001
DBP, mmHg	83.91 (11.49)	81.09 (11.71)	71.17 (8.30)	69.03 (8.57)	<0.001
Gender					0.006
Male	266 (40.92%)	321 (49.38%)	292 (44.92%)	318 (48.92%)	
Female	384 (59.08%)	329 (50.62%)	358 (55.08%)	332 (51.08%)	
Drinking status					0.014
Current	145 (22.31%)	170 (26.15%)	176 (27.12%)	162 (25.00%)	
Never	450 (69.23%)	428 (65.85%)	440 (67.80%)	456 (70.37%)	
Quit	55 (8.46%)	52 (8.00%)	33 (5.08%)	30 (4.63%)	
Smoking status					<0.001
Never	439 (67.54%)	372 (57.23%)	419 (64.46%)	387 (59.54%)	
Current	141 (21.69%)	220 (33.85%)	168 (25.85%)	225 (34.62%)	
Quit	70 (10.77%)	58 (8.92%)	63 (9.69%)	38 (5.85%)	
Education, n (%)					0.004
Below primary	315 (48.46%)	341 (52.46%)	294 (45.23%)	322 (49.54%)	
Primary schools	143 (22.00%)	153 (23.54%)	145 (22.31%)	140 (21.54%)	
Middle school	140 (21.54%)	124 (19.08%)	134 (20.62%)	137 (21.08%)	
High school and above	52 (8.00%)	32 (4.92%)	77 (11.85%)	51 (7.85%)	
Marital status					<0.001
Married	566 (87.08%)	538 (82.77%)	596 (91.69%)	572 (88.00%)	
Other	84 (12.92%)	112 (17.23%)	54 (8.31%)	78 (12.00%)	
Hypertension					<0.001
No	0 (0.00%)	32 (4.92%)	634 (97.54%)	633 (97.38%)	
Yes	650 (100.00%)	618 (95.08%)	16 (2.46%)	17 (2.62%)	
CVD					<0.001
Yes	124 (19.20%)	89 (13.80%)	70 (10.82%)	49 (7.66%)	
No	522 (80.80%)	556 (86.20%)	577 (89.18%)	591 (92.34%)	
Stroke					0.016
Yes	23 (3.55%)	17 (2.63%)	11 (1.69%)	7 (1.09%)	
No	624 (96.45%)	630 (97.37%)	638 (98.31%)	638 (98.91%)	

Values were expressed as mean (standard deviation) or medians (quartile interval) or n (%). Abbreviations: WC, waist circumference; BMI, body mass index; SBP, systolic blood pressure; DBP, diastolic blood pressure; HbA1c, hemoglobin A1c; HDL‐C, high‐density lipoprotein‐cholesterol; LDL‐C, low‐density lipoprotein‐cholesterol; TC, total cholesterol; TG, triglycerides; UA, uric acid; BUN, blood urea nitrogen; Cr, creatinine; CVD, cardiovascular disease; eGDR, estimated glucose disposal rate.

### Baseline characteristics according to glycemic metabolic status during follow-up

**Figure 2:**
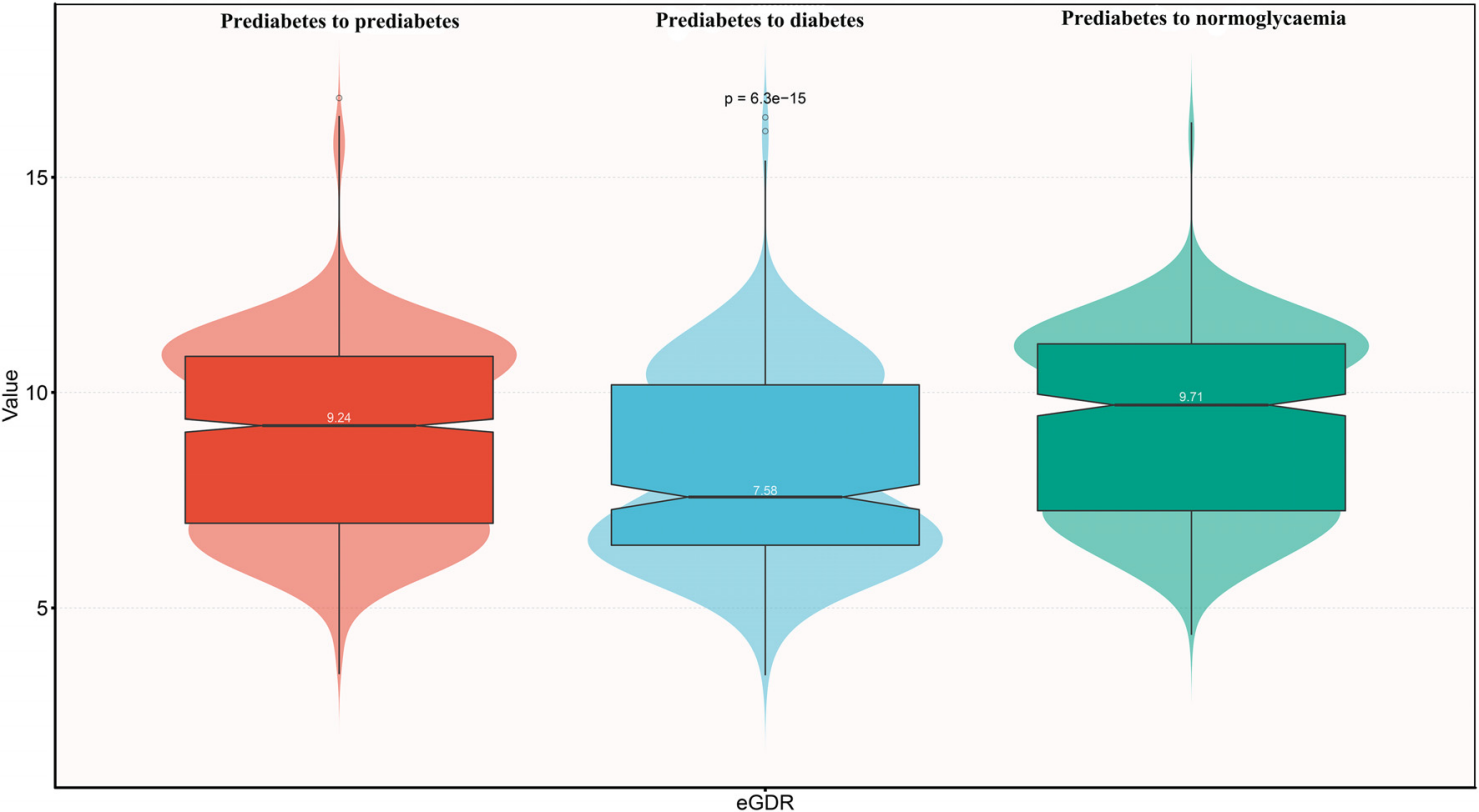
Violin chart showing baseline characteristics of eGDR according to glucose status during follow-up. eGDR, estimated glucose disposal rate.

During the median follow-up period of 4 years, 399 participants progressed to diabetes, 586 reverted to normoglycaemia, and 1,615 remained in the prediabetic state. As summarized in **Table 3**, participants who progressed to diabetes were generally older, less educated, more likely to be female, and had higher comorbid hypertension, higher levels of weight, WC, BMI, UA, TC, TG, glucose, HbA1c, SBP, and DBP, as well as lower levels of height, Cr, HDL-C, and eGDR (**Figure 2**). Participants who reverted to normoglycaemia showed opposite characteristics.

**Table 3:** Baseline characteristics summarized according to subjects’ glycemic status during follow-up.

	Glucose status during follow-up			*P*-value
	Prediabetes	Diabetes	Normoglycaemia	
No. of subjects	1615	399	586	
Age, years	59.65 (8.66)	60.62 (8.96)	58.32 (8.85)	<0.001
Height, m	1.58 (0.09)	1.57 (0.08)	1.59 (0.08)	<0.001
Weight, kg	59.08 (11.11)	61.65 (12.20)	59.37 (11.23)	<0.001
WC, cm	84.88 (12.39)	88.30 (12.76)	83.96 (10.92)	<0.001
BMI, kg/m^2^	23.64 (3.79)	25.07 (4.27)	23.46 (3.77)	<0.001
Cr, mg/dL	0.76 (0.66-0.88)	0.75 (0.63-0.86)	0.77 (0.67-0.88)	0.075
UA, mg/dL	4.49 (1.22)	4.55 (1.30)	4.46 (1.28)	0.547
TC, mg/dL	197.55 (173.97-222.68)	197.55 (175.90-225.00)	187.89 (166.62-211.08)	<0.001
TG, mg/dL	109.74 (77.44-161.07)	115.94 (84.96-182.31)	105.31 (75.22-155.76)	0.005
HDL-C, mg/dL	49.87 (41.17-61.08)	46.39 (37.50-57.80)	49.87 (40.59-60.70)	<0.001
LDL-C, mg/dL	120.23 (98.58-143.04)	119.07 (99.55-144.20)	110.18 (89.30-134.15)	<0.001
Glucose, mmol/L	6.00 (0.42)	6.16 (0.49)	5.98 (0.40)	<0.001
HbA1c, %	5.25 (0.41)	5.40 (0.43)	5.05 (0.36)	<0.001
SBP, mmHg	131.25 (21.25)	132.95 (19.21)	129.23 (21.08)	0.021
DBP, mmHg	76.21 (11.94)	77.17 (11.81)	75.82 (12.02)	0.215
eGDR	9.24 (6.97-10.84)	7.58 (6.46-10.18)	9.71 (7.26-11.13)	<0.001
Gender				<0.001
Male	729 (45.14%)	157 (39.35%)	311 (53.07%)	
Female	886 (54.86%)	242 (60.65%)	275 (46.93%)	
Drinking status				0.234
Current	412 (25.51%)	83 (20.85%)	158 (27.05%)	
Never	1096 (67.86%)	286 (71.86%)	392 (67.12%)	
Quit	107 (6.63%)	29 (7.29%)	34 (5.82%)	
Smoking status				0.473
Never	1012 (62.66%)	255 (63.91%)	350 (59.73%)	
Current	470 (29.10%)	107 (26.82%)	177 (30.20%)	
Quit	133 (8.24%)	37 (9.27%)	59 (10.07%)	
Education, n (%)				0.043
Below primary	806 (49.91%)	209 (52.38%)	257 (43.86%)	
Primary schools	352 (21.80%)	91 (22.81%)	138 (23.55%)	
Middle school	337 (20.87%)	66 (16.54%)	132 (22.53%)	
High school and above	120 (7.43%)	33 (8.27%)	59 (10.07%)	
Marital status				0.743
Married	1414 (87.55%)	344 (86.22%)	514 (87.71%)	
Other	201 (12.45%)	55 (13.78%)	72 (12.29%)	
Hypertension				<0.001
No	824 (51.02%)	160 (40.10%)	315 (53.75%)	
Yes	791 (48.98%)	239 (59.90%)	271 (46.25%)	
CVD				0.016
Yes	197 (12.27%)	68 (17.30%)	67 (11.55%)	
No	1408 (87.73%)	325 (82.70%)	513 (88.45%)	
Stroke				<0.001
Yes	29 (1.80%)	19 (4.81%)	10 (1.72%)	
No	1581 (98.20%)	376 (95.19%)	573 (98.28%)	

Values were expressed as mean (standard deviation) or medians (quartile interval) or n (%). Abbreviations: WC, waist circumference; BMI, body mass index; SBP, systolic blood pressure; DBP, diastolic blood pressure; HbA1c, hemoglobin A1c; HDL‐C, high‐density lipoprotein‐cholesterol; LDL‐C, low‐density lipoprotein‐cholesterol; TC, total cholesterol; TG, triglycerides; UA, uric acid; BUN, blood urea nitrogen; Cr, creatinine; CVD, cardiovascular disease; eGDR, estimated glucose disposal rate.

### Association Between eGDR and prediabetes progression or reversion

As shown in **Table 4**, both before and after adjusting for covariates, eGDR remained negatively associated with the progression of prediabetes to diabetes and positively associated with the reversion of prediabetes to normoglycaemia. In the crude model, each unit increase in eGDR was associated with a 14% reduction in the likelihood of progressing to diabetes (HR=0.86, 95% CI: 0.82, 0.90) and a 7% increase in the likelihood of reverting to normoglycaemia (HR=1.07, 95% CI: 1.03, 1.11). According to the results of Model III, each unit increase in eGDR was associated with a 19% reduction in the likelihood of progressing to diabetes (HR=0.81, 95% CI: 0.70, 0.93) and a 14% increase in the likelihood of reverting to normoglycaemia (HR=1.14, 95% CI: 1.05, 1.23). Additionally, when eGDR was converted from a continuous variable to a categorical variable and included in the Cox regression model, similar results were observed, with higher eGDR being negatively associated with the progression of prediabetes and positively associated with its reversion.

**Table 4:** Multivariate Cox regression analysis of the role of eGDR in assessing changes in glycemic status in patients with prediabetes.

	No. of case	HR (95%CI)			
		Non-adjusted Model	Model I	Model II	Model III
Prediabetes to normoglycaemia					
eGDR		1.07 (1.03, 1.11)	1.05 (1.00, 1.09)	1.15 (1.07, 1.24)	1.14 (1.05, 1.23)
eGDR(quartiles)					
Q1	108 (16.62%)	Ref	Ref	Ref	Ref
Q2	160 (24.62%)	1.48 (1.16, 1.89)	1.53 (1.16, 2.00)	1.60 (1.20, 2.12)	1.55 (1.16, 2.06)
Q3	130 (20.00%)	1.20 (0.93, 1.55)	1.16 (0.89, 1.51)	1.52 (0.87, 2.68)	1.52 (0.86, 2.68)
Q4	188 (28.92%)	1.74 (1.37, 2.21)	1.70 (1.27, 2.27)	2.25 (1.26, 4.04)	2.22 (1.23, 4.00)
*P*-trend		<0.0001	0.0231	<0.0001	0.0001
Prediabetes to Diabetes					
eGDR		0.86 (0.82, 0.90)	0.91 (0.86, 0.96)	0.80 (0.69, 0.92)	0.81 (0.70, 0.93)
eGDR(quartiles)					
Q1	149 (22.92%)	Ref	Ref	Ref	Ref
Q2	99 (15.23%)	0.66 (0.52, 0.86)	0.82 (0.62, 1.09)	0.73 (0.54, 1.00)	0.79 (0.58, 1.08)
Q3	94 (14.46%)	0.63 (0.49, 0.82)	0.76 (0.58, 0.99)	0.40 (0.19, 0.81)	0.40 (0.20, 0.83)
Q4	57 (8.77%)	0.38 (0.28, 0.52)	0.54 (0.38, 0.76)	0.25 (0.12, 0.56)	0.26 (0.12, 0.58)
*P*-trend		<0.0001	0.0005	0.0005	0.0015

HR, hazard ratios; CI, confidence interval; other abbreviations as in **Table 2**.

Model I adjusted for age, gender, married status, height, BMI.

Model II adjusted for age, gender, married status, height, BMI, SBP, DBP, drinking status, smoking status, education, marital status, CVD, stroke.

Model III adjusted for age, gender, married status, height, BMI, SBP, DBP, drinking status, smoking status, education, marital status, CVD, stroke, Cr, UA, TG, HDL-C, LDL-C, Glucose.

### Dose-response relationship between eGDR and prediabetes progression or reversion

**Figure 3:**
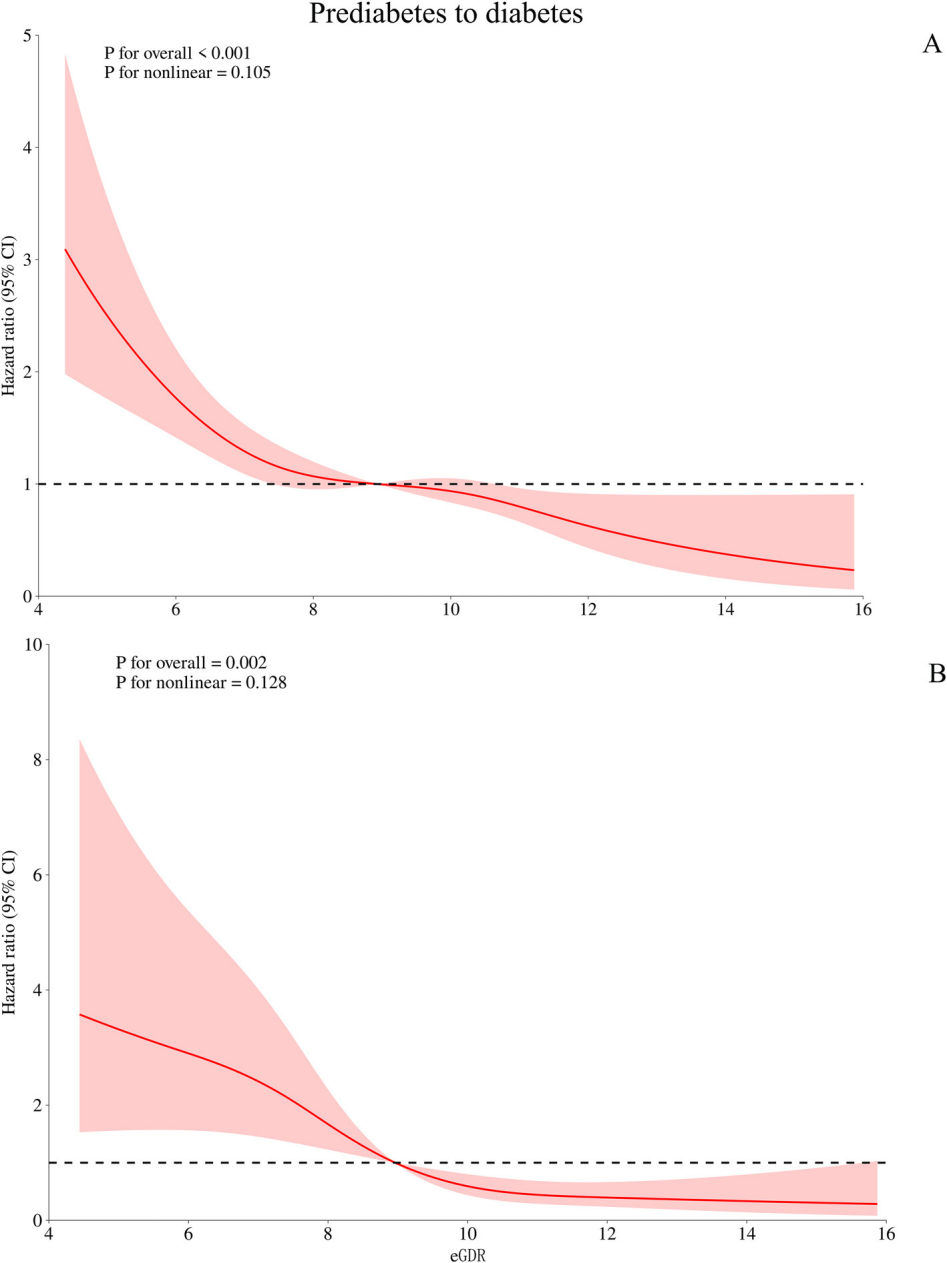
Visualizing the relationship between eGDR and progression from prediabetes to diabetes using 4-knots RCS (**A:** unadjusted; **B:** adjusted). eGDR, estimated glucose disposal rate.

**Figure 4:**
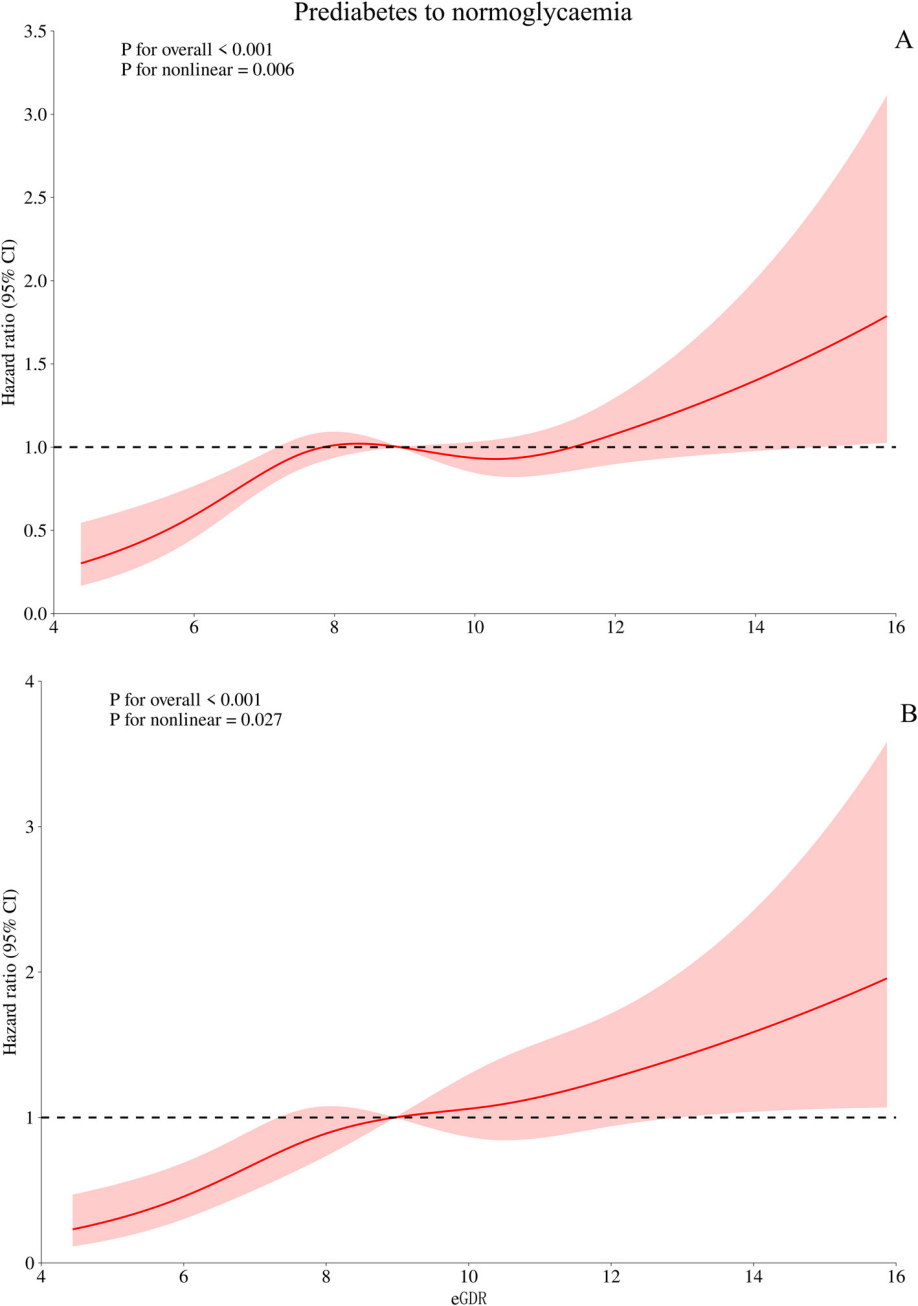
Visualizing the relationship between eGDR and regression of prediabetes to normoglycaemia using 4-knots RCS (**A**: unadjusted; **B**: adjusted). eGDR, estimated glucose disposal rate.

Based on Model III, we further used RCS to fit the dose-response relationship between eGDR and the progression or reversion of prediabetes. As shown in **Figure 3**, the relationship between eGDR and the risk of progression from prediabetes to diabetes was linear (*P* for non-linearity > 0.05), with a decreasing risk of progression as eGDR increased. In contrast, as shown in **Figure 4**, the relationship between eGDR and the reversion of prediabetes to normoglycaemia was nonlinear, resembling an L-shape (*P* for non-linearity < 0.05), with a potential threshold effect. Specifically, before the inflection point, the rate of reversion to normoglycaemia increased more rapidly with higher eGDR, whereas after the inflection point, the rate of reversion increased at a slower pace. Using a recursive algorithm, we calculated the inflection point value associated with reversion to normoglycaemia as 6.81 (**Table 5**). When the eGDR value was below 6.81, the rate of reversion to normoglycaemia was faster (HR: 1.98, 95% CI: 1.38, 2.84, *P* for log-likelihood ratio test = 0.0002), consistent with the RCS results.

**Table 5:** The result of the two-piecewise Cox regression model.

	HR (95%CI)	*P*-value
Prediabetes to normoglycaemia		
Fitting model by two-piecewise cox regression		
The inflection point of eGDR	6.81	
<6.81	1.98 (1.38, 2.84)	0.0002
>6.81	1.10 (1.01, 1.19)	0.0300
*P* for log likelihood ratio test		0.001

HR, hazard ratios; CI, confidence;

Adjusted for age, gender, married status, height, BMI, SBP, DBP, drinking status, smoking status, academic degree, marital status, heart disease, stroke, Cr, UA, TG, HDL-C, LDL-C, Glucose.

### Subgroup analysis

**Figure 5:**
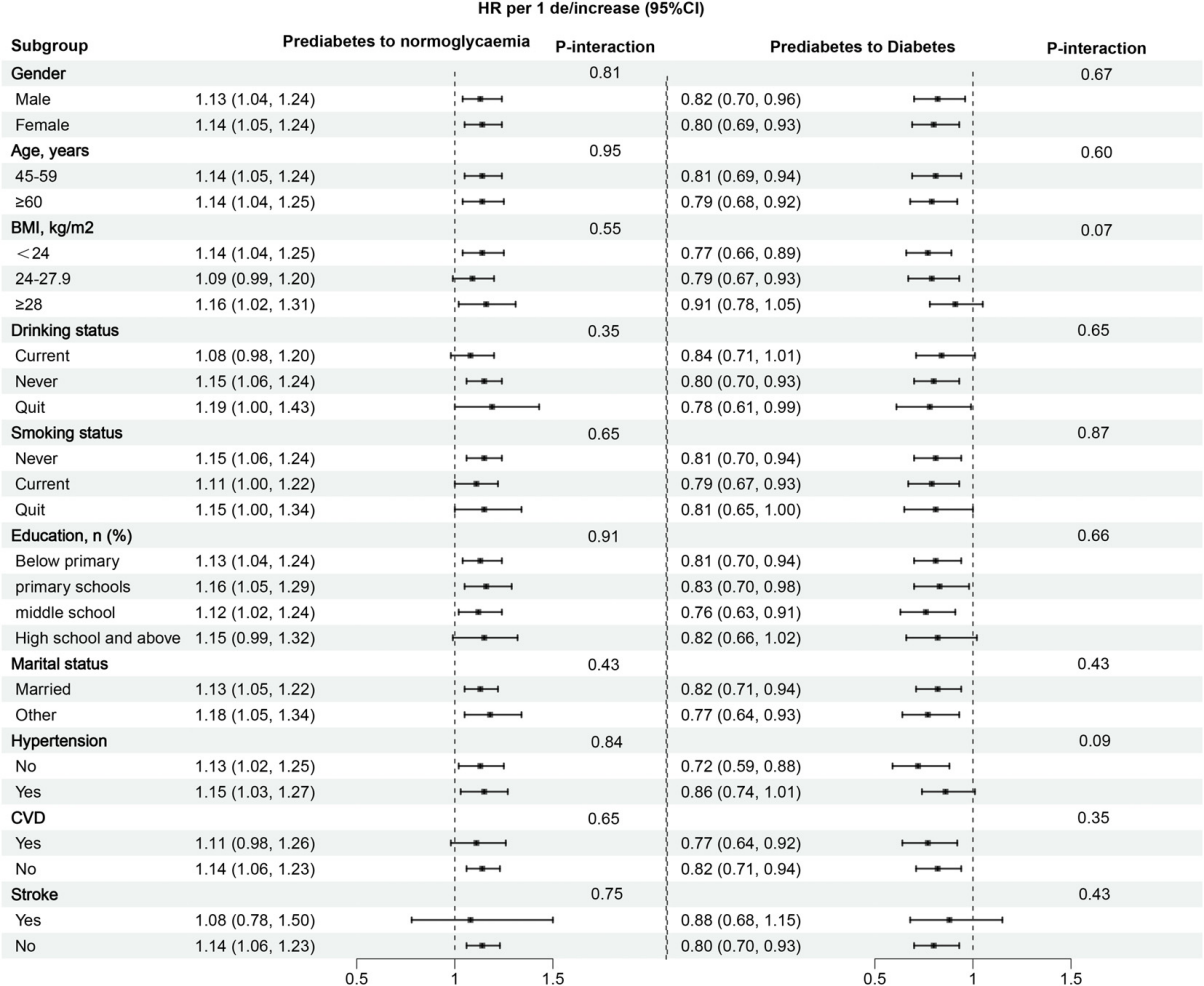
Subgroup analysis of the role and differences of eGDR in assessing changes in glycemic status in prediabetes patients. eGDR, estimated glucose disposal rate. BMI, body mass index; CVD, cardiovascular disease.

To further validate whether the study results were population-dependent, we conducted stratified analyses by gender, age, BMI, drinking/smoking status, education, marital status, hypertension, CVD, and stroke. The results showed no significant differences across all subgroups (all *P*-interactions > 0.05) (**Figure 5**). These findings suggested that the association between eGDR and the progression or reversion of prediabetes is relatively robust.

### Sensitivity analysis

In several sensitivity analyses, the results showed no significant changes (**Supplementary Table S3**). First, the main analysis was repeated after changing the inclusion criteria based on the Chinese population’s diagnostic standards, and no significant changes were observed. Next, using the Fine-Gray method to account for competing risks, we repeated the same analysis steps, and the results did not change substantially. Additionally, the main analysis was conducted after adjusting for the quadratic term of age, with no significant changes in the results. Finally, after imputing missing data, the results remained unchanged.

## Discussion

This nationwide prospective study investigated the association between a surrogate marker of insulin resistance (IR)-eGDR-and the reversion or progression of prediabetes among middle-aged and elderly individuals in China. The findings suggest that higher eGDR is positively associated with the reversion of prediabetes to normoglycaemia and negatively associated with the progression to diabetes. Additionally, the study identified a potential threshold effect point in the dose-response curve between eGDR and the reversion of prediabetes to normoglycaemia, with a HR of 1.98 associated with eGDR values less than 6.81.

The natural course of prediabetes includes maintaining the prediabetic state, progressing to diabetes, or reverting to normoglycaemia (28). A significant body of literature has previously reported a close association between poor glycemic metabolism and adverse outcomes (1, 29–31). Therefore, early identification of factors influencing the progression and reversion of diabetes is crucial for disease prevention and prognosis. IR plays a critical role in the progression of diabetes and is also a key factor in the reversion of prediabetes (12, 13, 32, 33). Previous studies have described associations between certain IR surrogates and the reversion of prediabetes, such as the triglyceride glucose index, triglycerides glucose and body mass index, and the metabolic score for IR (34–36). These findings further confirm the important application value of surrogate biomarkers of IR in glucose metabolism disorders. However, it is important to note that most of these previous IR surrogates primarily considered lipid and glucose factors, overlooking other metabolic factors. eGDR is a novel IR assessment indicator that integrates evaluations of obesity, glucose, and blood pressure factors. Previous studies have shown that eGDR has similar accuracy to the HEGC in assessing IR status (14). Recent studies further validate the significant role of eGDR in evaluating disease prognosis and outcomes. However, most eGDR-related studies to date have focused on analyzing complications or severe adverse outcomes in diabetic patients (15–18, 37, 38). Currently, there is limited understanding of the relationship between eGDR and prediabetes, a stage where glucose levels are still modifiable, and the association of eGDR with the reversion of prediabetes to normoglycaemia remains unclear. To address this issue, the current study analyzed data from the Chinese CHARLS cohort, revealing that higher eGDR promotes the reversion of prediabetes to normoglycaemia. After fully adjusting for confounding factors, the probability of reversion to normoglycaemia in the high eGDR group (Q4) was 2.22 times higher than in the low eGDR group (Q1). This finding underscores the important evaluative value of eGDR in the regression of prediabetes and suggests that monitoring eGDR during glucose control or intervention in prediabetes may be significantly beneficial.

The relationship between IR and its surrogates with diabetes onset has been well documented in numerous previous studies (9–11, 39–41). However, the association between the new IR surrogate eGDR and diabetes was unclear. To clarify this relationship, the current study further analyzed the association between eGDR and diabetes onset in prediabetic patients. After a median follow-up of 4 years, 399 participants (15.3%) in our study cohort progressed to diabetes. The association analysis results showed that after controlling for confounding factors, eGDR was significantly negatively associated with the progression of prediabetes to diabetes. Further analysis based on eGDR quartiles revealed that the risk of progressing to diabetes in the high eGDR group (Q4) was 74% lower than in the low eGDR group (Q1). Contrary to the findings of previous studies on IR (9–11, 39–41), where IR is typically positively associated with diabetes, eGDR in this study was negatively associated with diabetes. This finding suggests that high eGDR inhibits the progression of prediabetes. Notably, the negative association between eGDR and disease has also been reported in recent observational studies, including those on vascular-related diseases (17–19, 37), heart failure (42), atherosclerosis (43, 44), and various chronic complications of diabetes (37, 38, 45, 46). Furthermore, high eGDR has been identified as a protective factor against adverse outcomes such as atrial fibrillation recurrence and mortality (15–17, 40, 44, 47). These findings collectively indicate that maintaining low eGDR has detrimental effects on health, and early intervention is recommended for individuals with low eGDR. Although there are no studies yet on the mechanism by which low eGDR leads to the progression of prediabetes to diabetes, the pathophysiology of IR suggests that increased IR during the transition from normoglycaemia to prediabetes leads to β-cell dysfunction; when β-cell function cannot overcome IR, progression from prediabetes to diabetes occurs (32, 33, 48). As eGDR increases, representing a reduction in IR, the progression of prediabetes is suppressed (14). Considering the current study’s context and findings, we recommend dynamic monitoring of eGDR in prediabetic patients and maintaining this value at a high level whenever possible.

In the RCS analysis, we observed an intriguing finding: a nonlinear, L-shaped relationship between eGDR and the reversion of prediabetes to normoglycaemia. Further analysis calculated the eGDR threshold associated with reversion to normoglycaemia as 6.81. Specifically, when the eGDR level was below 6.81, each unit increase in eGDR was associated with a 98% increase in the likelihood of prediabetes reversion (HR: 1.98, 95% CI: 1.38-2.84, *P* = 0.0002); when the eGDR level was above 6.81, the likelihood of prediabetes reversion significantly decreased and stabilized (HR: 1.10, 95% CI: 1.01-1.19, *P* = 0.03). Similar L-shaped associations have also been reported in recent studies. In a recent study of non-alcoholic fatty liver disease (NAFLD) patients, Song et al. identified an eGDR threshold of 5.95 as a potential cutoff point for NAFLD patients at risk of developing atherosclerosis. When eGDR was below 5.95, the probability of atherosclerosis accelerated in NAFLD patients (43). Additionally, a study from Switzerland on mortality reported similar results; Nyström et al. found an L-shaped relationship between eGDR and all-cause mortality in patients with type 1 diabetes, with a threshold between 8-10, where the risk of all-cause mortality stabilized when eGDR exceeded the threshold (16). Based on these findings and the current study results, we believe that attention should be paid to evaluating the nonlinear relationship of eGDR in metabolic-related diseases.

“Treating pre-disease” is an important concept in Traditional Chinese Medicine (49, 50), where “pre-disease” refers to a transitional state before the onset of illness. Prediabetes is an early stage of diabetes as well as multiple chronic complications. From both public health and clinical perspectives, identifying and recognizing this intermediate-risk group is essential, as early intervention in the prediabetic phase is most effective in preventing diabetes, cardiovascular diseases, and various chronic complications (3–8). Therefore, timely diagnosis of the prediabetic population and effective management of key modifiable factors are crucial for preventing chronic complications. Against this backdrop, the findings of the current study hold significant implications for clinical practice: (1) As a non-invasive and simple surrogate for IR, eGDR offers notable advantages over the HEGC in clinical practice, substantially reducing both the economic and time costs for patients. (2) According to the findings of the current study, eGDR provides significant assistance in assessing the transition of glucose status; in summary, a low eGDR is detrimental to the restoration of normal glucose levels in prediabetes and may also accelerate the progression to diabetes. Clinicians can utilize eGDR to evaluate the trends and potential for glucose progression and reversal in patients with prediabetes. (3) Although the mechanism underlying the relationship between eGDR and glycemic metabolic transitions remains unclear, analyzing the development background and constituent components of eGDR suggests that preventing hypertension and obesity can be of significant assistance in promoting glycemic control in prediabetes. (4) The findings of the current study can also provide references for the construction of future prediction models for the progression or regression of prediabetes. Incorporating eGDR may further enhance the predictive performance of these models.

## Strengths and limitations

Strengths: (1) This study is the first to describe the association between eGDR and the natural course of prediabetes, with novel findings related to both progression to diabetes and reversion to normoglycemia. (2) The study includes a large, nationally representative cohort and validates its findings across different standards and through sensitivity analyses.

Limitations: (1) Although the study controlled for a large number of confounding factors in the analysis, it cannot eliminate the potential bias from unmeasured or residual confounders (51). (2) The data collected in this study partly relied on self-reporting, supported by a review of existing medical records, but did not include systematic adjudication of clinical outcomes, which may introduce some recall bias (52). (3) The results of this study are based on a middle-aged and elderly Chinese population, which may differ in genetic background compared to younger populations and Western adults. In the future, results verification will be required in young people and other races. (4) The study’s broad geographic follow-up led to some loss of follow-up, resulting in missing data. (5) Due to the lack of oral glucose tolerance test data in the CHARLS cohort, in the current study, we primarily relied on the definition of impaired fasting glucose in the American Diabetes Association standards to diagnose prediabetes. This may have resulted in an underestimation of the incidence rates of prediabetes and diabetes, as well as the regression rate of normoglycemia in the study; additionally, the missing data on impaired glucose tolerance prevented further exploration of the differences in the association between eGDR and glycemic metabolism. Future research needs to further differentiate between subtypes of prediabetes to provide more accurate evidence. (6) In the current study, the specific diagnosis time of prediabetes in the study population could not be determined, making it impossible to ascertain the duration of prediabetes in the patients. This may have a certain impact on the assessment of glycemic transition. Future studies are recommended to further analyze the influence of the duration of prediabetes on subsequent glycemic transition. (7) The follow-up period of this study remains relatively short, making it uncertain whether the association between eGDR and glycemic transition in prediabetes remains stable over a longer follow-up duration. It is recommended to conduct further studies with a longer follow-up period and to explore the impact of changes in eGDR during follow-up on glycemic transition. (8) The current study has only established the association between the IR surrogate eGDR and glycemic transition; Considering that multiple non-invasive IR surrogates have already been developed (34–36), it is necessary to conduct comparative studies in the future to further determine which IR surrogate is most suitable for the assessment of glycemic transition.

## Conclusion

In this prospective cohort study of middle-aged and elderly Chinese individuals, our results support a close association between higher eGDR and an increased likelihood of prediabetes reversion to normoglycaemia, as well as a decreased likelihood of progression to diabetes.

## Data Availability

The raw data supporting the conclusions of this article will be made available by the authors, without undue reservation.
