# Supplementary material for: Association between estimated glucose disposal rate and prediabetes reversion and progression: a nationwide cohort study of middle-aged and elderly people in China
Source: Front Endocrinol (Lausanne). 2025 Mar 21;16:1500993. doi: 10.3389/fendo.2025.1500993 (PMC11968371; doi:10.3389/fendo.2025.1500993)
Supplement: Supplementary file 1 [file DataSheet1.pdf]

Association Between Estimated Glucose Disposal Rate and Prediabetes Reversion and Progression: A nationwide cohort study of middle-aged and elderly people in China

Supplemental Methods

Study designs of the CHARLS

The China Health and Retirement Longitudinal Study (CHARLS) was a prospective cohort study conducted in China. In wave 1, a nationally representative sample of 17,708 participants was recruited from 28 provinces in 2011 via multistage probability sampling. The primary aim of this study was to recruit participants aged ≥ 45 years, but some participants aged 40 to 44 years also attended the baseline survey. All 17,708 participants underwent face-to-face interviews by the trained staff using the standardized questionnaire to collect data on sociodemographic information, lifestyles, and health-related information. Among 17,708 participants, 13,978 participants conducted anthropometric measurements to collect data on height, weight, waist circumference, blood pressure, grip strength, and so on. In addition, 11,847 participants provided blood samples for the laboratory test. Biochemical indicators, including total cholesterol, high-density lipoprotein cholesterol, low-density lipoprotein cholesterol, glycated hemoglobin, fasting blood glucose, and C-reactive protein were measured by the blood test. The follow-up surveys were conducted in 2013 (wave 2), 2015 (wave 3), and 2018 (wave 4) with questionnaire interviews and anthropometric measurements. Blood samples were collected in 2015 again, and biochemical indicators were measured by the blood test.

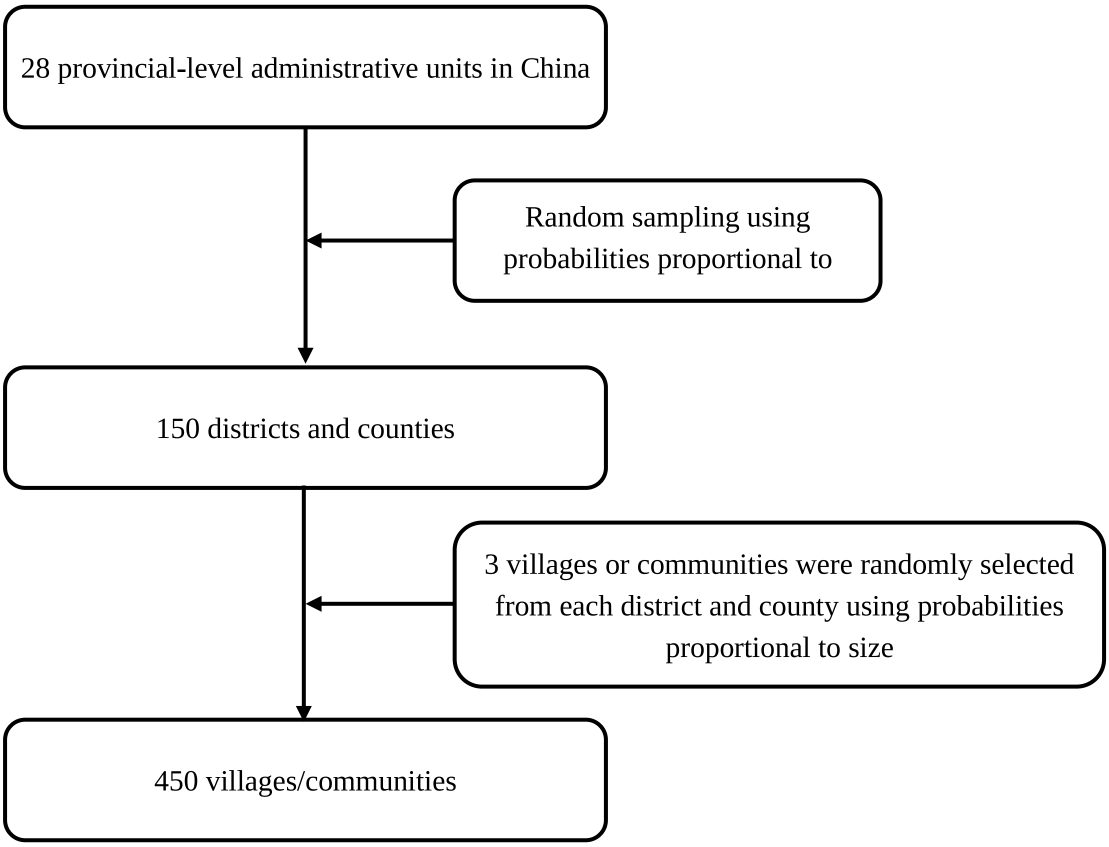

Supplementary Figure 1: The selection process of CHARLS.

Ascertainment of cardiovascular disease, stroke and hypertension

In the CHARLS, cardiovascular disease (CVD) was ascertained based on the self-reported physician-diagnosed heart disease. In each wave of the cohort, participants were asked "Have you been told by a doctor that you have been diagnosed with a heart disease, including angina, heart attack, congestive heart failure, and other heart problems?"

Likewise, strokes were determined by doctors based on patient self-reports,In each wave of the cohort, participants were asked "Have you been told by a doctor that you have been diagnosed with a stroke?".

Hypertension was clinically diagnosed when systolic blood pressure ≥ 140 mmHg and/or diastolic blood pressure ≥ 90 mmHg or the present use of antihypertensive drugs.
