# Supplementary material for: Association between estimated glucose disposal rate and prediabetes reversion and progression: a nationwide cohort study of middle-aged and elderly people in China
Source: Front Endocrinol (Lausanne). 2025 Mar 21;16:1500993. doi: 10.3389/fendo.2025.1500993 (PMC11968371; doi:10.3389/fendo.2025.1500993)
Supplement: Supplementary file 2 [file Table1.pdf]

Supplementary Table 1: Collinearity screening of eGDR and covariates.

	VIF			
	Step 1	Step 2	Step 3	Step 4
Age	1.6	1.6	1.6	1.6
Gender	3	3	2.9	2.9
Drinking status	1.1	1.1	1.1	1.1
Smoking status	1.6	1.6	1.6	1.6
Education	1.3	1.3	1.3	1.3
Marry status	1.1	1.1	1.1	1.1
Hypertension	Inf	NA	NA	NA
CVD	1	1	1	1
Stroke	1	1	1	1
Cr	1.7	1.7	1.7	1.7
UA	1.6	1.6	1.6	1.6
TC	17.3	17.3	17.3	NA
TG	6	6	6	1.5
HDL-C	3.8	3.8	3.8	1.5
LDL-C	14.6	14.6	14.6	1.1
Glucose	1.1	1.1	1.1	1.1
HbA1c	9007199254740992	1.1	1.1	1.1
SBP	2.8	2.8	2.8	2.8
DBP	2.3	2.3	2.3	2.3
Height	30.8	30.8	2	2
Weight	89.7	89.7	NA	NA
BMI	63.5	63.5	1.9	1.9
WC	Inf	2.6	2.6	2.6
eGDR	Inf	3.2	3.2	3.2

Abbreviations: Inf: infinity; VIF: Variance inflation factor; Other abbreviations as in Table 2.

Note-1: Variance inflation factor = $1/(1-R^2)$. Abbreviations as in Table 1.

Note-2: The variables with Variance inflation factor >5 will be regarded as collinear variables and cannot be included in the multiple regression model.

Supplemental Table 2. The missing number and rate of covariates

	Non- Missing	Missing
Age	2600	0
Gender	2600	0
Drinking status	2597	3
Smoking status	2600	0
Education	2600	0
Marry status	2600	0
Hypertension	2600	0
CVD	2578	22
Stroke	2588	12
Cr	2600	0
UA	2600	0
TC	2600	0
TG	2600	0
HDL-C	2600	0
LDL-C	2599	1
Glucose	2600	0
HbA1c	2600	0
SBP	2568	32
DBP	2568	32
Height	2591	9
Weight	2595	5
BMI	2588	12
WC	2600	0
eGDR	2600	0

Abbreviations as in Table 2.

Supplementary Table 3: Sensitivity analysis.

Sensitivity-1	HR (95%CI)
Prediabetes to normoglycaemia	
eGDR	1.14 (1.08, 1.20)
Prediabetes to Diabetes	
eGDR	0.84 (0.78, 0.89)
Sensitivity-2	SHR (95%CI)
Prediabetes to normoglycaemia	
eGDR	1.08 (1.04, 1.12)
Prediabetes to Diabetes	
eGDR	0.84 (0.79, 0.88)
Sensitivity-3	HR (95%CI)
Prediabetes to normoglycaemia	
eGDR	1.14 (1.06, 1.23)
Prediabetes to Diabetes	
eGDR	0.80 (0.69, 0.92)
Sensitivity-4	HR (95%CI)
Prediabetes to normoglycaemia	
eGDR	1.13 (1.05, 1.22)
Prediabetes to Diabetes	
eGDR	0.76 (0.68, 0.85)

SHR: subdistribution hazard ratios; CI: confidence interval; other abbreviations as in Table 1.

Note 1: Models adjusted for the same covariates as in model III (Table 4).

Note 2: (1) Sensitivity-1: including 1,227 subjects according to Chinese expert consensus on prediabetes's diagnostic criteria for diabetes, prediabetes and normoglycaemia; (3) Sensitivity-2: Results of Competing Risks Model Analysis; (4) sensitivity-3: based on model III, with additional adjustments for the quadratic term of age; (4) sensitivity-4: Use the median to impute missing values and analyze them.
